# Reservoirs of Porcine Circoviruses: A Mini Review

**DOI:** 10.3389/fvets.2019.00319

**Published:** 2019-09-19

**Authors:** Shao-Lun Zhai, Shou-Sheng Lu, Wen-Kang Wei, Dian-Hong Lv, Xiao-Hui Wen, Qi Zhai, Qin-Ling Chen, Yan-Wei Sun, Yun Xi

**Affiliations:** ^1^Key Laboratory of Animal Disease Prevention of Guangdong Province, Animal Disease Diagnostic Center, Institute of Animal Health, Guangdong Academy of Agricultural Sciences, Scientific Observation and Experiment Station of Veterinary Drugs and Diagnostic Techniques of Guangdong Province, Ministry of Agriculture and Rural Affairs, Guangzhou, China; ^2^Guangdong Center for Animal Disease Prevention and Control, Guangzhou, China; ^3^Department of Clinical Laboratory, The Third Affiliated Hospital of Sun Yat-Sen University, Guangzhou, China

**Keywords:** porcine circovirus, PCV, PCV1, PCV2, PCV3, reservoirs

## Abstract

Porcine circovirus (PCV) is one of the smallest known DNA viruses in mammals. At present, PCVs are divided into three species, PCV1, PCV2, and PCV3. PCV1 and PCV2 were found in the 1970s and the 1990s, respectively, whereas PCV3 was discovered recently in 2016. PCV1 does not cause diseases in pigs. However, PCV3, similar to PCV2, is reported to be associated with several swine diseases, including porcine dermatitis and nephropathy syndrome (PDNS) and reproductive failure. PCVs are very common in domestic pigs as well as wild boars. However, PCVs have been occasionally isolated from non-porcine animals, including ruminants (such as cattle, goats, wild chamois, and roe deers), rodents (such as NMRI mice, BALB/c mice, Black C57 mice, ICR mice, *Mus musculus*, and *Rattus rattus*), canines (such as dogs, minks, foxes, and raccoon dogs), insects (such as flies, mosquitoes, and ticks), and shellfish. Moreover, PCVs are frequently reported in biological products, including human vaccines, animal vaccines, porcine-derived commercial pepsin products, and many cell lines. PCVs are also abundant in the environment, including water samples and air samples. Interestingly, PCV1 and/or PCV2 antibody or antigen has also been detected in sera, stool samples and respiratory swab samples of human, revealing zoonotic potential of PCVs. Thus, PCVs inhabit many types of reservoirs. In this review, we summarize the reservoirs of PCVs, and this information would be helpful in understanding the natural circulating status and possible cross-species transmission of PCVs.

## Introduction

Porcine circoviruses (PCVs) are members of the *Circovirus* genus of the *Circoviridae* family. Currently, there are three species in the genus, PCV type 1 (PCV1), PCV type 2 (PCV2), and PCV type 3 (PCV3), respectively. In 1974, PCV1 was discovered as a contaminant in porcine kidney cell lines (1). Subsequent studies have confirmed that PCV1 is apathogenic in pigs (2). In late 1990s, a new porcine disease, called post-weaning multisystemic wasting syndrome (PMWS), emerged in North America and Europe (3–6), and PCV2 was confirmed as the causal pathogen (7, 8). PCV2 has a global distribution and diverse genotypes (9, 10). In 2016, the third PCV, named PCV3, was discovered using high-throughput sequencing technology in U.S. swine herds suffering from porcine dermatitis and nephropathy syndrome (PDNS), reproductive failure and other syndromes (11, 12). A recent study suggested that PCV3 fulfilled Koch's postulates and could cause PDNS in piglets (13).

PCVs, especially PCV2 and PCV3, are very common in pigs, and can cause diverse clinical presentations including PMWS, PDNS, reproductive failure, interstitial pneumonia, and so on. Both, PCV2 and PCV3 have garnered immense interest in the world swine industry. Hitherto, most studies found in literature have focused on PCVs derived from swine sources. Occasionally, PCVs have also been isolated from non-porcine animals, biological products, and environmental samples.

## PCVs in Non-Porcine Animals

### PCVs in Ruminants

In 1995, for the first time, Tischer et al. confirmed the presence of PCV1 antibodies in cattle in Germany using indirect immunofluorescence assay (IFA) and enzyme-linked immunosorbent assay (ELISA) (14). Later, a PCV2 nucleotide (1,768 bp) was identified in cattle with respiratory diseases and aborted bovine fetuses in Canada (15). In 2007, a new disease called hemorrhagic diathesis broke out in calves in Germany, and PCV2b (1,767 bp) was suggested as a potential causal pathogen (16, 17). In the United States and China, PCV2 was frequently detected using metagenomic sequencing method and special PCR method in beef from supermarkets, beef stalls, and goat samples (18–22). Interestingly, one previous study confirmed that calves were susceptible to PCV2 and presented lymph node swelling, reddening of oral and ocular mucosa, and diarrhea post inoculation (23).

In 2011, a circovirus-like virus sequence (PorkNW2, GenBank accession number HQ738638) was discovered in both pork and beef samples from stores in San Francisco (18). In 2014, two similar sequences were re-detected in beef samples from the Sunset district of San Francisco (19). In fact, these circovirus-like virus sequences were significantly close to the aforementioned PCV3 in pigs (11, 12). Based on these research reports, it is speculated that PCV3 is also widespread in cattle. A recent epidemiological investigation from China confirmed our speculation. Their results showed that 74 out of 213 (34.7%) clinically healthy bovine samples from eight regions in the Shandong Province were positive for PCV3, and that the bovine-origin PCV3 genome sequences had a close relationship with porcine-origin PCV3 genome sequences (24). Surprisingly, PCV3 was also reported in the samples of wild chamois and roe deers (25). The data suggest that ruminants, especially cattle, are noteworthy reservoirs of PCVs. These epidemiological data bring to light two important scientific issues that warrant further research: (1) to determine whether PCV3 has a multi-host receptor and (2) to identify whether some cross-species transmission routes (such as direct contact, insect biting) might contribute to the circulation of PCV3 in pigs and ruminants.

### PCVs in Rodents

Seroconversion of PCV1 antibody was detected in several murine species including NMRI, BALB/c, and Black C57 (14). Based on evidence from numerous studies, mice are often considered important animal models of PCV2 infection (26–35). However, only a few studies have conformed PCV infection in field rodent samples. Since 2010, several research teams from Hungary, Brazil, Korea and China have provided molecular evidence of PCV2 in rodents circulating on swine farms (36–39). In the study from Brazil, PCV2 antigen was identified in the spleen, lungs and kidneys of rodent species *Mus musculus* and *Rattus rattus* using immunohistochemistry assay (37). In addition, rodent-origin PCV2 genome sequences had remarkable similarity with gene sequences of PCV2 isolated from pigs (37, 39). It is noteworthy that one recent study identified PCV3 in commercially sourced laboratory mice including BALB/c and ICR mice (40). This is in contrast with from the Hungarian study, which claims that rodents need certain contact with pigs, without which PCV2 is negative in rodents (36). Owing to limited information, we are currently unable to ascertain the origin of murine PCV3 in laboratory mice. Without significant clinical manifestation, rodents are more of a carrying reservoir and an animal model of PCVs. On farms, unconstrained rodents may promote the spread of PCVs.

### PCVs in Canines

Fur animals have significant economic value. In recent years, cases of infections due to porcine pathogens (such as Hepatitis E virus, Pseudorabies virus) have progressively in minks and foxes (41–44). PCV2 was also reported in minks, foxes, and raccoon dogs with diarrhea or reproductive failure in China (45–47). Interestingly, the samples were tested negative for common pathogens (such as Mink enteritis virus, Canine distemper virus, Coronavirus, Rotavirus, or Astrovirus) of minks and foxes, indicating that PCV2 was also an important pathogen contributing to the clinical diseases of minks and foxes. Infection of PCV2 in fur animals may be caused by consumption of food containing porcine-origin products and possibly by cross-species transmission. These mechanisms could also explain PCV2 and PCV3 infection in dogs (48, 49).

### PCVs in Insects

Generally speaking, PCV is not considered as an arbovirus. However, PCV2 has been often detected in *M. domestica* flies and culex mosquitoes in pig farms (50, 51). Surprisingly, PCV3 was first identified in ticks (*Ixodes ricinus*) collected from wild roe deers that were negative for PCV3, making the source of PCV3 ambiguous (25). PCV infection is postulated to occur when hosts get bitten by carrier flies, mosquitoes, and ticks.

### PCVs in Mussels

Bivalve shellfishes (such as oysters, clams, and mussels) intake nutrition by filtering up to 4.8 L/h of surrounding water and simultaneously concentrate microorganisms that are present (52–54). Animal pathogens can contaminate the beds via runoff from fields fertilized using animal waste. One previous study reported high detection rate for PCV2 and *E. coli* (41%, 12/29 and 28%, 8/29) in blue mussels (*Mytilus edulis*) from Danish commercial harvesting areas, while common food-borne pathogens (such as hepatitis E virus, rotavirus, *Salmonella*) were absent. The geographic distribution of the PCV2-positive shellfish samples revealed that positive samples were localized to Limfjord in the northern part of Jutland and a bay area in the south-western part of Jutland, Denmark (55). This suggested that these bay areas were contaminated by porcine waste, and PCV2 may be a specific indicator of porcine waste in shellfish.

## PCVs in Human Samples

There has always been controversy regarding PCVs infecting human. At the outset, Tischer et al. confirmed the presence of PCV1 antibodies in human sera, and the IFA results showed a significantly higher number of positive sera (20%) in non-hospitalized “healthy” persons from the former German Democratic Republic than that (8.6%) in blood donors from Berlin-West (14). Interestingly, scientists from Northern Ireland, the United States, and Germany could not detect antibodies or antigens of PCV2 in human samples (56–59). However, in other studies, antibody of PCV1 or antigen of PCV2 was detected in human sera, digestive tract samples and respiratory tract samples (60–65). Although there is serological and molecular proof of PCV presence in humans, the detection rate is very low. Thus, the clinical significance of PCVs in human beings remains largely unknown.

## PCVs in Biological Products

PCV1 was first confirmed as a contaminant in porcine kidney cell lines (1), following which, non-infectious PCV1 and PCV2 were detected in porcine-derived commercial pepsin products used for humans (66). Owing to its porcine origin, the detection of PCVs in pepsin and other porcine biological products is perfectly plausible. PCV1 was also reported in commercial veterinary vaccines against classic swine fever virus (CSFV), porcine parvovirus (PPV), and pseudorabies virus (PRV) (67, 68). However, in 2010, PCV1 was reported in human rotavirus vaccine (69), which caused a significant stir because of unknown clinical significance of PCVs in humans. Since that incident, the Food and Drug Administration (FDA) and several research institutions began investigating the source of the vaccine contamination and assessing the safety of the vaccine. Their results indicated that several cell lines, virus seeds and vaccines were contaminated by PCV1 and/or PCV2 (70–75). Porcine-derived commercial pepsin product used for cell culture and vaccine production was considered as the main source of vaccine contamination (76). Although the contaminated vaccines caused no clinical diseases in people, humans can be considered likely reservoirs for PCVs (63, 77). Thus, via human fecal matter, PCVs might have been introduced into environmental water or other places (64).

## PCVs in Environmental Samples

As we know, many viruses can spread through aerosols. Whether PCVs can spread via aerosol is little known. However, Canadian researchers found that high viral loads (up to 10^7^ genomes per cubic meter of air) of PCV2 existed in swine confinement buildings (78). Another study also identified the existence of PCV2 in bioaerosol samples from pig farms and abattoirs. In addition, PCV2 was detected in nasal washes of workers (4/78) from pig farms (65). This revealed that PCV2 was a potential airborne virus.

Intriguingly, PCV2 can be detected in various types of water samples from Brazil, including (a) water used for swine consumption, subjected to conventional treatment followed by chlorination; (b) tap water meant for human consumption, subjected to conventional treatment followed by chlorination; (c) surface water without treatment, used for swine consumption; (d) water from the Pinhal river, which crosses; (e) groundwater collected from a tap located at a school; and (f) water from the Jacutinga river, which is redirected and treated for domestic supply (79). This indicated that PCV2 was widely prevalent in the environment.

## Conclusions and Discussion

In summary, from the current knowledge, we infer that PCVs have multiple reservoirs; they are not limited to the swine family, but have broad distributed in ruminants, rodents, canines, insects, and other species. Consequently, we deduce the occurrence of possible cross-species transmission (such as pigs to rodents, pigs to cattle, pigs to fur animals) of PCVs. In most cases of detection or infection in non-porcine animals, the main reason has been ingestion of porcine products or direct contact with pigs (36–39, 45, 47, 51). Cases of infection of PCVs in ruminants are not well-evaluated, because ruminants hardly ingest porcine products or have rare or no contact with pigs. Due to the wide distribution of PCVs in various types of water samples (79), there is a high risk of infection in ruminants via PCV-contaminated water. In fact, owing to their wide tissue tropism, PCVs could exist in almost all tissues of pigs and wild boars (80, 81). Furthermore, PCVs were frequently detected in fecal samples of non-porcine animals (45, 48, 62, 63). Discharge of PCV-positive fecal matter into water bodies increases the occurrence of PCV contamination. A comprehensive understanding of PCV reservoirs would be invaluable for implementing accurate measures to control the spread of PCVs.

## Author Contributions

S-LZ: conceptualization and writing—original draft preparation. S-SL, W-KW, D-HL, X-HW, QZ, Q-LC, and Y-WS: writing—review and editing. S-LZ and YX: supervision. S-LZ and W-KW: project administration. All of authors have approved this manuscript for publication.

### Conflict of Interest

The authors declare that the research was conducted in the absence of any commercial or financial relationships that could be construed as a potential conflict of interest.

## References

[1] Tischer I Rasch R Tochtermann G. Characterization of papovavirus-and picornavirus-like particles in permanent pig kidney cell lines. Zentralbl Bakteriol Orig A. (1974) 226:153–67. 4151202

[2] Tischer I Mields W Wolff D Vagt M Griem W. Studies on epidemiology and pathogenicity of porcine circovirus. Arch Virol. (1986) 91:271–6. 10.1007/BF01314286 3778212

[3] Allan GM McNeilly F Kennedy S Daft B Clarke EG Ellis JA. Isolation of porcine circovirus-like viruses from pigs with a wasting disease in the USA and Europe. J Vet Diagn Invest. (1998) 10:3–10. 10.1177/104063879801000102 9526853

[4] Allan GM Mc Neilly F Meehan BM Kennedy S Mackie DP Ellis JA. Isolation and characterisation of circoviruses from pigs with wasting syndromes in Spain, Denmark and Northern Ireland. Vet Microbiol. (1999) 66:115–23. 10.1016/S0378-1135(99)00004-8 10227473

[5] Ellis J Hassard L Clark E Harding J Allan G Willson P. Isolation of circovirus from lesions of pigs with postweaning multisystemic wasting syndrome. Can Vet J. (1998) 39:44–51. 9442952 PMC1539838

[6] Wellenberg GJ Pesch S Berndsen FW Steverink PJ Hunneman W Van der Vorst TJ. Isolation and characterization of porcine circovirus type 2 from pigs showing signs of post-weaning multisystemic wasting syndrome in The Netherlands. Vet Q. (2000) 22:167–72. 10.1080/01652176.2000.9695049 10952449

[7] Ellis J Krakowka S Lairmore M Haines D Bratanich A Clark E. Reproduction of lesions of postweaning multisystemic wasting syndrome in gnotobiotic piglets. J Vet Diagn Invest. (1999) 11:3–14. 10.1177/104063879901100101 9925205

[8] Kennedy S Moffett D McNeilly F Meehan B Ellis J Krakowka S Reproduction of lesions of postweaning multisystemic wasting syndrome by infection of conventional pigs with porcine circovirus type 2 alone or in combination with porcine parvovirus. J Comp Pathol. (2000) 122:9–24. 10.1053/jcpa.1999.0337 10627387

[9] Zhai SL Chen SN Xu ZH Tang MH Wang FG Li XJ. Porcine circovirus type 2 in China: an update on and insights to its prevalence and control. Virol J. (2014) 11:88. 10.1186/1743-422X-11-88 24885983 PMC4031328

[10] Franzo G Segalés J. Porcine circovirus 2 (PCV-2) genotype update and proposal of a new genotyping methodology. PLoS ONE. (2018) 13:e0208585. 10.1371/journal.pone.0208585 30521609 PMC6283538

[11] Palinski R Piñeyro P Shang P Yuan F Guo R Fang Y. A novel porcine circovirus distantly related to known circoviruses is associated with porcine dermatitis and nephropathy syndrome and reproductive failure. J Virol. (2016) 91:e01879–16. 10.1128/JVI.01879-16 27795441 PMC5165205

[12] Phan TG Giannitti F Rossow S Marthaler D Knutson TP Li L. Detection of a novel circovirus PCV3 in pigs with cardiac and multi-systemic inflammation. Virol J. (2016) 13:184. 10.1186/s12985-016-0642-z 27835942 PMC5105309

[13] Jiang H Wang D Wang J Zhu S She R Ren X. Induction of porcine dermatitis and nephropathy syndrome in piglets by infection with Porcine circovirus type 3. J Virol. (2019) 93:e02045–18. 10.1128/JVI.02045-18 30487279 PMC6363995

[14] Tischer I Bode L Apodaca J Timm H Peters D Rasch R. Presence of antibodies reacting with porcine circovirus in sera of humans, mice, and cattle. Arch Virol. (1995) 140:1427–39. 10.1007/BF01322669 7544971

[15] Nayar GP Hamel AL Lin L Sachvie C Grudeski E Spearman G. Evidence for circovirus in cattle with respiratory disease and from aborted bovine fetuses. Can Vet J. (1999) 40:277–8. 10200889 PMC1539691

[16] Kappe EC Halami MY Schade B Alex M Hoffmann D Gangl A. Bone marrow depletion with haemorrhagic diathesis in calves in Germany: characterization of the disease and preliminary investigations on its aetiology. Berl Munch Tierarztl Wochenschr. (2010) 123:31–41. 10.2376/0005-9366-123-31 20135908

[17] Halami MY Müller H Böttcher J Vahlenkamp TW. Whole-genome sequences of two strains of porcine circovirus 2 isolated from calves in Germany. Genome Announc. (2014) 2:e01150–13. 10.1128/genomeA.01150-13 24407647 PMC3886960

[18] Li L Shan T Soji OB Alam MM Kunz TH Zaidi SZ. Possible cross-species transmission of circoviruses and cycloviruses among farm animals. J Gen Virol. (2011) 92(Pt 4):768–72. 10.1099/vir.0.028704-0 21177928 PMC3133700

[19] Zhang W Li L Deng X Kapusinszky B Delwart E What is for dinner? Viral metagenomics of US store bought beef, pork, and chicken. Virology. (2014) 468–70:303–10. 10.1016/j.virol.2014.08.025 PMC4252299 25217712

[20] Zhai SL Chen RA Chen SN Wen XH Lv DH Wu DC. First molecular detection of porcine circovirus type 2 in bovids in China. Virus Genes. (2014) 49:507–11. 10.1007/s11262-014-1117-1 25248785

[21] Zhai SL He DS Qi WB Chen SN Deng SF Hu J. Complete genome characterization and phylogenetic analysis of three distinct buffalo-origin PCV2 isolates from China. Infect Genet Evol. (2014) 28:278–82. 10.1016/j.meegid.2014.10.005 25460822

[22] Wang X Li W Xu X Wang W He K Fan H. Phylogenetic analysis of two goat-origin PCV2 isolates in China. Gene. (2018) 651:57–61. 10.1016/j.gene.2018.01.095 29408624

[23] Halami MY Freick M Shehata AA Müller H Vahlenkamp TW. Susceptibility of calves to porcine circovirus-2 (PCV2). Vet Microbiol. (2014) 173:125–31. 10.1016/j.vetmic.2014.06.022 25085519

[24] Wang W Sun W Cao L Zheng M Zhu Y Li W. An epidemiological investigation of porcine circovirus 3 infection in cattle in Shandong province, China. BMC Vet Res. (2019) 15:60. 10.1186/s12917-019-1793-0 30760271 PMC6375139

[25] Franzo G Grassi L Tucciarone CM Drigo M Martini M Pasotto D. A wild circulation: high presence of *Porcine circovirus* 3 in different mammalian wild hosts and ticks. Transbound Emerg Dis. (2019) 66:1548–57. 10.1111/tbed.13180 30901142

[26] Kiupel M Stevenson GW Choi J Latimer KS Kanitz CL Mittal SK. Viral replication and lesions in BALB/c mice experimentally inoculated with porcine circovirus isolated from a pig with postweaning multisystemic wasting disease. Vet Pathol. (2001) 38:74–82. 10.1354/vp.38-1-74 11199167

[27] Quintana J Balasch M Segalés J Calsamiglia M Rodríguez-Arrioja GM Plana-Durán J. Experimental inoculation of porcine circoviruses type 1 (PCV1) and type 2 (PCV2) in rabbits and mice. Vet Res. (2002) 33:229–37. 10.1051/vetres:2002011 12056474

[28] Kiupel M Stevenson GW Galbreath EJ North A HogenEsch H Mittal SK. Porcine circovirus type 2 (PCV2) causes apoptosis in experimentally inoculated BALB/c mice. BMC Vet Res. (2005) 1:7. 10.1186/1746-6148-1-7 16259631 PMC1291377

[29] Cságola A Cadar D Tuboly T. Replication and transmission of porcine circovirus type 2 in mice. Acta Vet Hung. (2008) 56:421–7. 10.1556/AVet.56.2008.3.15 18828493

[30] Opriessnig T Patterson AR Jones DE Juhan NM Meng XJ Halbur PG. Limited susceptibility of three different mouse (*Mus musculus*) lines to Porcine circovirus-2 infection and associated lesions. Can J Vet Res. (2009) 73:81–6. 19436587 PMC2666323

[31] Li J Yuan X Zhang C Miao L Wu J Shi J. A mouse model to study infection against porcine circovirus type 2: viral distribution and lesions in mouse. Virol J. (2010) 7:158. 10.1186/1743-422X-7-158 20630105 PMC2920255

[32] Deng ZB Wang ND Xu DJ Yuan AW Ge M Luo W. Viral distribution and lesions in Kunming mice experimentally infected with porcine circovirus type 2b. Vet Res Commun. (2011) 35:181–92. 10.1007/s11259-011-9461-2 21287271

[33] Deng ZB Yuan AW Luo W Wang ND Gong QL Yu XL. Transmission of porcine circovirus type 2b (PCV2b) in Kunming mice. Acta Vet Hung. (2013) 61:234–43. 10.1556/AVet.2013.004 23661391

[34] Liu G Yang G Guan G Zhang Y Ren W Yin J. Effect of dietary selenium yeast supplementation on porcine circovirus type 2 (PCV2) infections in mice. PLoS ONE. (2015) 10:e0115833. 10.1145/2818302 25723390 PMC4344303

[35] de Castro AM Cruz TF Yamada KB Gerber PF Gabardo MP Araújo JP Jr. Preliminary evidence of age-dependent clinical signs associated with porcine circovirus 2b in experimentally infected CH3/Rockefeller mice. Res Vet Sci. (2015) 103:70–2. 10.1016/j.rvsc.2015.09.008 26679798

[36] Lorincz M Cságola A Biksi I Szeredi L Dán A Tuboly T. Detection of porcine circovirus in rodents - short communication. Acta Vet Hung. (2010) 58:265–8. 10.1556/AVet.58.2010.2.12 20460225

[37] Pinheiro AL Bulos LH Onofre TS de Paula Gabardo M de Carvalho OV Fausto MC. Verification of natural infection of peridomestic rodents by PCV2 on commercial swine farms. Res Vet Sci. (2013) 94:764–8. 10.1016/j.rvsc.2012.10.006 23141170

[38] Truong QL Seo TW Yoon BI Kim HC Han JH Hahn TW. Prevalence of swine viral and bacterial pathogens in rodents and stray cats captured around pig farms in Korea. J Vet Med Sci. (2013) 75:1647–50. 10.1292/jvms.12-0568 23892461 PMC3942947

[39] Zhai SL Chen SN Liu W Li XP Deng SF Wen XH. Molecular detection and genome characterization of porcine circovirus type 2 in rats captured on commercial swine farms. Arch Virol. (2016) 161:3237–44. 10.1007/s00705-016-3004-7 27530112

[40] Jiang S Zhou N Li Y An J Chang T. Detection and sequencing of porcine circovirus 3 in commercially sourced laboratory mice. Vet Med Sci. (2019) 5:176–81. 10.1002/vms3.144 30779321 PMC6498514

[41] Jin HL Gao SM Liu Y Zhang SF Hu RL. Pseudorabies in farmed foxes fed pig offal in Shandong province, China. Arch Virol. (2016) 161:445–8. 10.1007/s00705-015-2659-9 26563317

[42] Krog JS Breum SØ Jensen TH Larsen LE. Hepatitis E virus variant in farmed mink, Denmark. Emerg Infect Dis. (2013) 19:2028–30. 10.3201/eid1912.130614 24274600 PMC3840851

[43] Wang L Gong W Fu H Li M Zhang Y Luo Z. Hepatitis E virus detected from Chinese laboratory ferrets and farmed mink. Transbound Emerg Dis. (2018) 65:e219–23. 10.1111/tbed.12720 28941157

[44] Xie XT Macdonald RE Tapscott B Nagy E Turner PV. Detection of astrovirus, rotavirus C, and hepatitis E viral RNA in adult and juvenile farmed mink (*Neovison vison*). Front Vet Sci. (2018) 5:132. 10.3389/fvets.2018.00132 29974054 PMC6020771

[45] Wang GS Sun N Tian FL Wen YJ Xu C Li J. Genetic analysis of porcine circovirus type 2 from dead minks. J Gen Virol. (2016) 97:2316–22. 10.1099/jgv.0.000529 27324162

[46] Song T Zhang S Hao J Xin S Hui W Tang M. First detection and genetic analysis of fox-origin porcine circovirus type 2. Transbound Emerg Dis. (2019) 66:1–6. 10.1111/tbed.13004 30153367

[47] Song T Hao J Zhang R Tang M Li W Hui W. First detection and phylogenetic analysis of porcine circovirus type 2 in raccoon dogs. BMC Vet Res. (2019) 15:107. 10.1186/s12917-019-1856-2 30961660 PMC6454600

[48] Herbst W Willems H. Detection of virus particles resembling circovirus and porcine circovirus 2a (PCV2a) sequences in feces of dogs. Res Vet Sci. (2017) 115:51–3. 10.1016/j.rvsc.2017.01.014 28135670 PMC7111833

[49] Zhang J Liu Z Zou Y Zhang N Wang D Tu D. First molecular detection of porcine circovirus type 3 in dogs in China. Virus Genes. (2018) 54:140–4. 10.1007/s11262-017-1509-0 28983774

[50] Blunt R McOrist S McKillen J McNair I Jiang T Mellits K. House fly vector for porcine circovirus 2b on commercial pig farms. Vet Microbiol. (2011) 149:452–5. 10.1016/j.vetmic.2010.11.019 21145672

[51] Yang X Hou L Ye J He Q Cao S Detection of porcine circovirus type 2 (PCV2) in mosquitoes from pig farms by PCR. Pak Vet J. (2012) 32:134–5.

[52] Carver CEA Mallet AL Estimating the carrying capacity of a coastal inlet for mussel culture. Aquaculture. (1990) 88:39–53. 10.1016/0044-8486(90)90317-G

[53] Winter JE The filtration rate of *Mytilus edulis* and its dependence on algal concentration, measured by a continuous automatic recording apparatus. Mar Biol. (1973) 22:317–28. 10.1007/BF00391388

[54] Burkhardt W III Calci KR. Selective accumulation may account for shellfish-associated viral illness. Appl Environ Microbiol. (2000) 66:1375–8. 10.1128/AEM.66.4.1375-1378.2000 10742214 PMC91995

[55] Krog JS Larsen LE Schultz AC. Enteric porcine viruses in farmed shellfish in Denmark. Int J Food Microbiol. (2014) 186:105–9. 10.1016/j.ijfoodmicro.2014.06.012 25016209

[56] Allan GM McNeilly F McNair I Curran MD Walker I Ellis J Absence of evidence for porcine circovirus type 2 in cattle and humans, and lack of seroconversion or lesions in experimentally infected sheep. Arch Virol. (2000) 145:853–7. 10.1007/s007050050678 10893163

[57] Hattermann K Maerz A Slanina H Schmitt C Mankertz A. Assessing the risk potential of porcine circoviruses for xenotransplantation: consensus primer-PCR-based search for a human circovirus. Xenotransplantation. (2004) 11:547–50. 10.1111/j.1399-3089.2004.00181.x 15479465

[58] Burbelo PD Ragheb JA Kapoor A Zhang Y. The serological evidence in humans supports a negligible risk of zoonotic infection from porcine circovirus type 2. Biologicals. (2013) 41:430–4. 10.1016/j.biologicals.2013.09.005 24120888 PMC3838456

[59] Denner J Mankertz A. Porcine circoviruses and xenotransplantation. Viruses. (2017) 9:E83. 10.3390/v9040083 28425928 PMC5408689

[60] Han HH Karkada N Jayadeva G Dubin G. Serologic response to porcine circovirus type 1 (PCV1) in infants vaccinated with the human rotavirus vaccine, Rotarix™: a retrospective laboratory analysis. Hum Vaccin Immunother. (2017) 13:237–44. 10.1080/21645515.2016.1231262 27657348 PMC5287324

[61] Bernstein CN Nayar G Hamel A Blanchard JF. Study of animal-borne infections in the mucosas of patients with inflammatory bowel disease and population-based controls. J Clin Microbiol. (2003) 41:4986–90. 10.1128/JCM.41.11.4986-4990.2003 14605128 PMC262476

[62] Li L Kapoor A Slikas B Bamidele OS Wang C Shaukat S. Multiple diverse circoviruses infect farm animals and are commonly found in human and chimpanzee feces. J Virol. (2010) 84:1674–82. 10.1128/JVI.02109-09 20007276 PMC2812408

[63] Esona MD Mijatovic-Rustempasic S Yen C Parashar UD Gentsch JR Bowen MD. Detection of PCV-2 DNA in stool samples from infants vaccinated with RotaTeq®. Hum Vaccin Immunother. (2014) 10:25–32. 10.4161/hv.26731 24104203 PMC4181015

[64] Mijatovic-Rustempasic S Immergluck LC Parker TC Laghaie E Mohammed A McFadden T. Shedding of porcine circovirus type 1 DNA and rotavirus RNA by infants vaccinated with Rotarix®. Hum Vaccin Immunother. (2017) 13:928–35. 10.1080/21645515.2016.1255388 27936349 PMC5404636

[65] Borkenhagen LK Mallinson KA Tsao RW Ha SJ Lim WH Toh TH. Surveillance for respiratory and diarrheal pathogens at the human-pig interface in Sarawak, Malaysia. PLoS ONE. (2018) 13:e0201295. 10.1371/journal.pone.0201295 30052648 PMC6063427

[66] Fenaux M Opriessnig T Halbur PG Xu Y Potts B Meng XJ. Detection and *in vitro* and *in vivo* characterization of porcine circovirus DNA from a porcine-derived commercial pepsin product. J Gen Virol. (2004) 85(Pt 11):3377–82. 10.1099/vir.0.80429-0 15483254

[67] Quintana J Segalés J Calsamiglia M Domingo M. Detection of porcine circovirus type 1 in commercial pig vaccines using polymerase chain reaction. Vet J. (2006) 171:570–3. 10.1016/j.tvjl.2004.12.008 16624728

[68] Wang C Pang VF Jeng CR Lee F Huang YW Lin YL. Detection of porcine circovirus type 1 in commercial porcine vaccines by loop-mediated isothermal amplification. Folia Microbiol (Praha). (2011) 56:483–9. 10.1007/s12223-011-0072-7 21948286 PMC3251781

[69] Victoria JG Wang C Jones MS Jaing C McLoughlin K Gardner S. Viral nucleic acids in live-attenuated vaccines: detection of minority variants and an adventitious virus. J Virol. (2010) 84:6033–40. 10.1128/JVI.02690-09 20375174 PMC2876658

[70] Ma H Shaheduzzaman S Willliams DK Gao Y Khan AS. Investigations of porcine circovirus type 1 (PCV1) in vaccine-related and other cell lines. Vaccine. (2011) 29:8429–37. 10.1016/j.vaccine.2011.07.123 21835219

[71] McClenahan SD Krause PR Uhlenhaut C. Molecular and infectivity studies of porcine circovirus in vaccines. Vaccine. (2011) 29:4745–53. 10.1016/j.vaccine.2011.04.087 21569811

[72] Baylis SA Finsterbusch T Bannert N Blümel J Mankertz A. Analysis of porcine circovirus type 1 detected in Rotarix vaccine. Vaccine. (2011) 29:690–7. 10.1016/j.vaccine.2010.11.028 21093497

[73] Gilliland SM Forrest L Carre H Jenkins A Berry N Martin J. Investigation of porcine circovirus contamination in human vaccines. Biologicals. (2012) 40:270–7. 10.1016/j.biologicals.2012.02.002 22402185

[74] Petricciani J Sheets R Griffiths E Knezevic I. Adventitious agents in viral vaccines: lessons learned from 4 case studies. Biologicals. (2014) 42:223–36. 10.1016/j.biologicals.2014.07.003 25135887

[75] Lackner C Leydold SM Modrof J Farcet MR Grillberger L Schäfer B. Reduction of spiked porcine circovirus during the manufacture of a Vero cell-derived vaccine. Vaccine. (2014) 32:2056–61. 10.1016/j.vaccine.2014.02.011 24560672

[76] Gagnieur L Cheval J Gratigny M Hébert C Muth E Dumarest M. Unbiased analysis by high throughput sequencing of the viral diversity in fetal bovine serum and trypsin used in cell culture. Biologicals. (2014) 42:145–52. 10.1016/j.biologicals.2014.02.002 24661556

[77] Dubin G Toussaint JF Cassart JP Howe B Boyce D Friedland L. Investigation of a regulatory agency enquiry into potential porcine circovirus type 1 contamination of the human rotavirus vaccine, Rotarix: approach and outcome. Hum Vaccin Immunother. (2013) 9:2398–408. 10.4161/hv.25973 24056737 PMC3981850

[78] Verreault D Létourneau V Gendron L Massé D Gagnon CA Duchaine C. Airborne porcine circovirus in Canadian swine confinement buildings. Vet Microbiol. (2010) 141:224–30. 10.1016/j.vetmic.2009.09.013 19773132

[79] Garcia LA Viancelli A Rigotto C Pilotto MR Esteves PA Kunz A. Surveillance of human and swine adenovirus, human norovirus and swine circovirus in water samples in Santa Catarina, Brazil. J Water Health. (2012) 10:445–52. 10.2166/wh.2012.190 22960488

[80] Segalés J. Porcine circovirus type 2 (PCV2) infections: clinical signs, pathology and laboratory diagnosis. Virus Res. (2012) 164:10–9. 10.1016/j.virusres.2011.10.007 22056845

[81] Ouyang T Niu G Liu X Zhang X Zhang Y Ren L. Recent progress on porcine circovirus type 3. Infect Genet Evol. (2019) 73:227–33. 10.1016/j.meegid.2019.05.009 31096019

